# Air quality standards for the concentration of particulate matter 2.5, global descriptive analysis

**DOI:** 10.2471/BLT.19.245704

**Published:** 2020-12-15

**Authors:** Yevgen Nazarenko, Devendra Pal, Parisa A Ariya

**Affiliations:** a Department of Atmospheric and Oceanic Sciences, McGill University, Montreal, Canada.; b Department of Chemistry, McGill University, 801 Sherbrooke Street West, Montréal, QC H3A 2K6, Canada.

## Abstract

**Objective:**

To compare ambient air quality standards for the mass concentration of aerosol particles smaller than approximately 2.5 μm (PM_2.5_) and exposure to these particles in national and regional jurisdictions worldwide.

**Methods:**

We did a review of government documents and literature on air quality standards. We extracted and summarized the PM_2.5_ concentration limits effective before July 2020, noting whether standards were enforced, voluntary or target. We compared averaging methods and permitted periods of time that standards may be exceeded. We made a descriptive analysis of PM_2.5_ standards by population, total area and population density of jurisdictions. We also compared data on actual PM_2.5_ air quality against the standards.

**Findings:**

We obtained data on standards from 62 jurisdictions worldwide, including 58 countries. Of the world’s 136.06 million km^2^ land under national jurisdictions, 71.70 million km^2^ (52.7%) lack an official PM_2.5_ air quality standard, and 3.17 billion people live in areas without a standard. The existing standards ranged from 8 to 75 µg/m^3^, mostly higher than the World Health Organization guideline annual limit of < 10 µg/m^3^. The weakest PM_2.5_ standards were often exceeded, while the more stringent standards were often met. Several jurisdictions with the highest population density demonstrated compliance with relatively stringent standards.

**Conclusion:**

The metrics used in PM_2.5_ ambient air quality standards should be harmonized worldwide to facilitate accurate assessment of risks associated with PM_2.5_ exposure. Population density alone does not preclude stringent PM_2.5_ standards. Modernization of standards can also include short-term standards to unmask PM_2.5_ fluctuations in high-pollution areas.

## Résumé

**Objectif:**

Comparer les normes de qualité de l'air ambiant en termes de concentration massique des particules en suspension dont le diamètre est inférieur à 2,5 μm environ (MP_2,5_) ainsi que l'exposition à ces particules dans les juridictions nationales et régionales du monde entier.

**Méthodes:**

Nous avons examiné les publications et documents officiels consacrés aux normes de qualité de l'air. Nous en avons extrait les limites de concentration en MP_2,5_ appliquées avant juillet 2020 et les avons synthétisées, en notant si ces normes étaient imposées, facultatives ou ciblées. Nous avons comparé les méthodes de calcul des moyennes et les périodes durant lesquelles il était possible de s'en écarter. Nous avons également réalisé une analyse descriptive des normes en matière de MP_2,5_ en fonction de la population, du territoire et de la densité démographique des juridictions. Enfin, nous avons effectué une comparaison entre les données concernant la qualité de l'air actuelle en termes de MP_2,5_ d'une part, et les normes de l'autre.

**Résultats:**

Nous avons obtenu des informations sur les normes en vigueur au sein de 62 juridictions à travers le monde, réparties dans 58 pays. Sur les 136,06 millions de km^2^ de territoires sous juridiction nationale, 71,70 millions de km^2^ (52,7%) ne faisaient l'objet d'aucune norme officielle fixant la qualité de l'air selon les MP_2,5_ et 3,17 milliards de personnes vivent dans des zones où il n'existe aucune norme en vigueur. Les normes actuelles vont de 8 à 75 µg/m^3^ et sont généralement supérieures à la limite annuelle < 10 µg/m^3^ définie dans les lignes directrices de l'Organisation mondiale de la Santé. Les normes MP_2,5_ les plus basses étaient fréquemment dépassées, tandis que les plus strictes étaient souvent respectées. De nombreuses juridictions affichant une forte densité démographique ont montré qu'elles se conformaient à des normes relativement strictes.

**Conclusion:**

Les chiffres employés pour déterminer les normes MP_2,5_ indiquant la qualité de l'air ambiant devraient être harmonisés dans le monde entier afin de mieux évaluer les risques associés à une exposition aux MP_2,5_. La densité démographique n'empêche pas à elle seule l'adoption de normes MP_2,5_ strictes. Par ailleurs, des mesures à court terme peuvent être intégrées dans la modernisation des normes pour identifier les fluctuations de MP_2,5_ dans les régions très polluées.

## Resumen

**Objetivo:**

Comparar las normas de calidad del aire ambiente en lo que respecta a la concentración en masa de partículas de aerosol inferiores a 2,5 μm (PM_2,5_) aproximadamente y la exposición a esas partículas en las jurisdicciones nacionales y regionales de todo el mundo.

**Métodos:**

Realizamos una revisión de los documentos del gobierno y la literatura sobre las normas de calidad del aire. Extrajimos y resumimos los límites de concentración de PM_2,5_ efectivos antes de julio de 2020, señalando si los estándares se aplicaban, eran voluntarios o eran objetivos. Comparamos los métodos de promediación y los períodos de tiempo permitidos en que se pueden superar los estándares. Hicimos un análisis descriptivo de los estándares de PM_2,5_ por población, superficie terrestre y densidad de población de las jurisdicciones. También comparamos los datos sobre la calidad real del aire de PM_2,5_ con los estándares.

**Resultados:**

Obtuvimos datos sobre las normas de 62 jurisdicciones de todo el mundo, incluidos 58 países. De los 136,06 millones de km^2^ del mundo que se encuentran bajo jurisdicciones nacionales, 71,70 millones de km^2^ (52,7%) carecen de un estándar oficial de calidad del aire de PM_2,5_, y 3,17 mil millones de personas viven en zonas sin estándar. Los estándares existentes oscilaban entre 8 y 75 µg/m^3^, en su mayoría superiores al límite anual de la Organización Mundial de la Salud de < 10 µg/m^3^. A menudo se superaban los estándares más débiles de PM_2,5_, mientras que a menudo se cumplían los estándares más estrictos. Varias jurisdicciones con la mayor densidad de población demostraron el cumplimiento de normas relativamente estrictas.

**Conclusión:**

Las mediciones utilizadas en las normas de calidad del aire ambiente de PM_2,5_ deben armonizarse en todo el mundo para facilitar la evaluación precisa de los riesgos asociados a la exposición a PM_2,5_. La densidad de población por sí sola no impide que se apliquen normas estrictas sobre las PM_2,5_. La modernización de las normas también puede incluir normas a corto plazo para desenmascarar las fluctuaciones de PM_2,5_ en áreas de alta contaminación.

## ملخص

الغرض مقارنة معايير جودة الهواء المحيط لتركيز كتلة جزيئات الهواء الأقل من 2.5 ميكرومتر تقريبًا (PM2.5)، والتعرض لهذه الجسيمات في السلطات القضائية الوطنية والإقليمية في جميع أنحاء العالم.

الطريقة قمنا بمراجعة الوثائق والمؤلفات الحكومية عن معايير جودة الهواء. قمنا باستخراج وتلخيص حدود تركيز جزيئات الهواء 2.5 ميكرومتر التي كانت سارية قبل يوليو/تموز 2020، مع ملاحظة ما إذا كانت المعايير إجبارية أو طوعية أو مستهدفة. قمنا بمقارنة طرق حساب المتوسط والفترات الزمنية المسموح بها والتي قد يتم تجاوز المعايير فيها. لقد أجرينا تحليلًا وصفيًا لمعايير 2.5 ميكرومتر حسب السكان، ومساحة الأرض، والكثافة السكانية للسلطات القضائية. قمنا أيضًا بمقارنة البيانات الخاصة بجودة الهواء بتركيز 2.5 ميكرومتر في مقابل المعايير.

النتائج حصلنا على بيانات بخصوص المعايير من 62 سلطة قضائية في حول العالم، بما يشمل 58 دولة. من بين 136.06 مليون كيلومتر مربع من الأراضي الخاضعة للسلطات القضائية الوطنية، تفتقر مساحة 71.70 مليون كيلومتر مربع (52.7%) إلى معيار رسمي لجودة الهواء بتركيز 2.5 ميكرومتر، ويعيش 3.17 مليار نسمة في مناطق دون معيار. تراوحت المعايير الحالية من 8 إلى 75 ميكروجرام/متر مكعب، وهي في الغالب أعلى من الحد السنوي لتوجيه منظمة الصحة العالمية، والبالغ أقل من 10 ميكروجرام/متر مكعب. تم غالبًا تجاوز أضعف معايير تركيز 2.5 ميكرومتر، بينما تم في الأغلب الوفاء بالمعايير الأكثر صرامة. أظهرت العديد من السلطات القضائية ذات الكثافة السكانية الأعلى امتثالها لمعايير صارمة نسبياً.

الاستنتاج يجب تنسيق المقاييس المستخدمة في معايير جودة الهواء المحيط بتركيز 2.5 ميكرومتر في جميع أنحاء العالم، وذلك لتسهيل التقييم الدقيق للمخاطر المرتبطة بالتعرض لتركيز 2.5 ميكرومتر. لا تعيق الكثافة السكانية وحدها المعايير الصارمة لتركيز 2.5 ميكرومتر. يمكن كذلك أن يشمل تحديث المعايير معايير قصيرة الأجل لكشف تقلبات التركيز 2.5 ميكرومتر في المناطق عالية التلوث.

## 摘要

**目的:**

旨在比较世界各国和各地区有关气溶胶颗粒【直径大约小于 2.5 微米 (PM_2.5_)】质量浓度和暴露情况的环境空气质量标准。

**方法:**

我们已查阅有关空气质量标准的政府文件和文献。我们提取并汇总了 2020 年 7 月前生效的 PM_2.5_ 浓度限值,并标明了各标准是要求强制、自愿还是有针对性地执行。我们比较了平均方法以及可能超出标准要求的允许时间段。基于辖区内的人口、土地面积和人口密度对 PM_2.5_ 标准进行了描述性分析。还将实际的 PM_2.5_ 空气质量数据与标准进行了比较。

**结果:**

我们采纳了全球 62 个司法管辖区(包括 58 个国家)与标准有关的数据。在全球 13,606 万平方公里受国家管辖的土地中,7,170 万平方公里(占 52.7%)缺乏正规的 PM_2.5_ 空气质量标准管制,31.7 亿人生活在未设置相关标准的地区。当前各标准的规定限值从 8 微克/立方米到 75 微克/立方米不等,大部分高于世界卫生组织发布的指南规定的年均浓度限值(低于 10 微克/立方米)。设置最宽松的 PM_2.5_ 标准,通常会超标,而若标准比较严格,反而通常会符合要求。调查显示,人口密度最高的几个司法管辖区采用了相对严格的标准。

**结论:**

PM_2.5_ 环境空气质量标准规定的指标应在全球范围内保持统一,以便准确评估 PM_2.5_ 暴露相关风险。不能仅因人口密度小而放弃采用严格的 PM_2.5_ 标准。标准的现代化改造还可能包括制定短期标准,以探明高污染地区的 PM_2.5_ 值波动情况。

## Резюме

**Цель:**

Сравнить стандарты качества окружающего воздуха для массовой концентрации аэрозольных частиц размером менее прибл. 2,5 мкм (PM_2,5_) и воздействие этих частиц на уровне государственных и региональных юрисдикций в мировом масштабе.

**Методы:**

Авторы выполнили критическую оценку официальных государственных документов и литературы по стандартам качества воздуха. Авторы извлекли и обобщили данные по предельной концентрации PM_2,5_, которая действовала до июля 2020 года, отмечая, были стандарты принудительными, добровольными или целевыми. Авторы сравнили методы усреднения и допустимые периоды времени, в течение которых стандарты могут быть превышены. Был выполнен описательный анализ стандартов PM_2,5_ по населению, площади территории и плотности населения соответствующих юрисдикций. Данные о фактическом качестве воздуха PM_2,5_ также сравнивались со стандартами.

**Результаты:**

Авторы получили данные по стандартам из 62 юрисдикций в мировом масштабе, включая 58 стран. Из 136,06 млн км^2^ территорий государственных юрисдикций в мире 71,70 млн км^2^ (52,7%) не имеют официального стандарта качества воздуха PM_2,5_, а 3,17 млрд человек проживают в районах, не имеющих стандарта. Существующие стандарты варьируются в диапазоне от 8 до 75 мкг/м^3^, что в большинстве случаев превышает годовой предел, установленный Всемирной организацией здравоохранения и составляющий <10 мкг/м^3^. Самые низкие стандарты PM_2,5_ часто превышались, в то время как более строгие стандарты часто соблюдались. Несколько юрисдикций с самой высокой плотностью населения демонстрировали соблюдение относительно строгих стандартов.

**Вывод:**

Показатели, используемые в стандартах качества окружающего воздуха PM_2,5_, необходимо согласовать во всемирном масштабе, чтобы обеспечить точную оценку рисков, связанных с воздействием PM_2,5_. Сама по себе плотность населения не препятствует соблюдению строгих стандартов PM_2,5_. Процесс совершенствования стандартов может также включать определение краткосрочных стандартов для выявления колебаний PM_2,5_ в районах с высокой степенью загрязнения воздуха.

## Introduction

Millions of people die prematurely every year due to cardiovascular disease, pulmonary disease and cancer caused by air pollution.^1^ For the premature deaths due to cancer, air pollution is a leading environmental cause.^2^ Pollutants in the air exist as gases, and solid and liquid airborne particles also called aerosols. Aerosols occur in wide-ranging sizes. Among the different metrics describing particle size, the most common is aerodynamic diameter (diameter of the spherical particle with a density of 1 g/m^3^ that has the same settling velocity as the given particle).^3^ Three particle size ranges with the upper limits of 10 μm, 2.5 μm and 1 μm are named PM_10_, PM_2.5_ and PM_1_, respectively. They are used to define fractions of aerosols for regulatory purposes. Only PM_10_ and PM_2.5_ are currently regulated in the form of ambient air quality standards. Of these two, we focus on PM_2.5_ due to its stronger association with adverse health effects.^1^

The PM_2.5_ component of air pollution was responsible for an estimated 4.2 million annual premature deaths globally in 2015.^4^ In 2010, China had 1.3 million premature deaths due to exposure to PM_2.5_, India had 575 000 and Pakistan had 105 000 deaths per year.^5^ The 28 European Union (EU) countries had 173 000 and the United States of America (USA) 52 000 annual premature deaths.^5^ Therefore, tightening and enforcing PM_2.5_ ambient air quality standards could reduce the burden of disease and premature mortality.

Here, we review PM_2.5_ standards worldwide and compare standards across different jurisdictions. 

## Methods

We carried out a review of PM_2.5_ air quality standards worldwide, following the applicable guidelines of Preferred Reporting Items for Systematic Review and Meta-Analysis Protocols (data repository).^6^

### Data sources

**Fig. 1:**
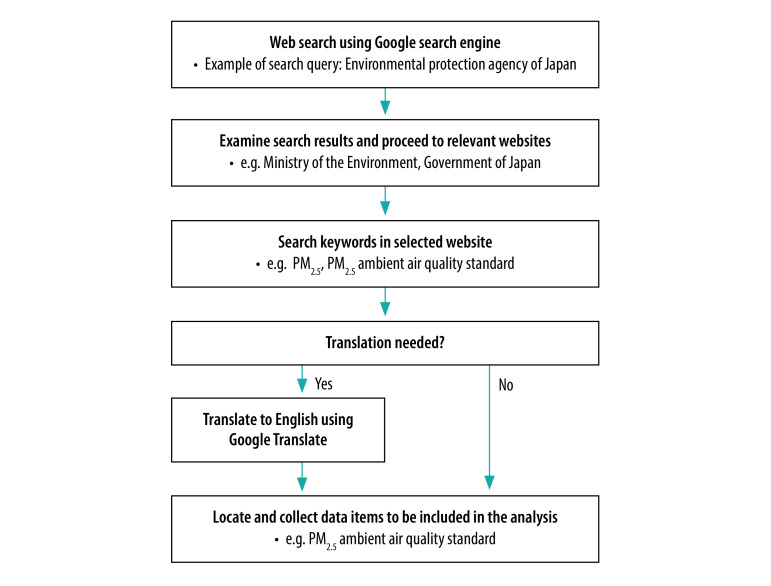
Search strategy for documents in the study of PM_2.5_ ambient air quality standards worldwide

We obtained the data on absolute particle mass concentration limits from regulatory documents, government websites and other sources published up to 27 October 2020. We used articles in peer-reviewed publications and documents of nationally or internationally recognized organizations when we were unable to identify government sources. We conducted an online search for each country listed in World Population Review,^7^ one by one, using the search strategy exemplified in Fig. 1 and described in detail in the data repository.^6^ Box 1 presents the eligibility criteria for inclusion in the analysis. We consulted documents in Arabic, English, French, Japanese, Korean, Mandarin, Persian, Russian, Spanish, Vietnamese and Ukrainian. We used Google Translate (Google LLC, Mountain View, USA) for some search strings, websites and documents. 

PM_2.5_: mass concentration of aerosol particles smaller than approximately 2.5 μm.

Note: We searched PM_2.5 _ambient air quality standards for individual countries listed in World Population Review.^7^ For the full search strategy see data repository.^6^

Box 1 **Eligibility criteria for inclusion of documents in the study of PM_2.5 _ambient air quality standards worldwide**

The standards had to be published in government documents, on government websites, in government-commissioned reports, reports of nationally or globally recognized organizations, or in peer-reviewed publications.

Documents in any official language were acceptable.

Eligible standards had to specifically mention PM_2.5_ or its equivalent in the language of the document or define the regulated fraction of ambient particulate air pollution as particles or aerosols smaller than approximately 2.5 µm. Conditions constituting a part of, or the full, ISO definition of PM_2.5_ were allowed.

Only annual and 24-hour standards were considered for the summary analysis. Standards with other averaging periods were included in the summary table only.

Multinational, national and regional jurisdictions were included. Self-determination by jurisdictions was sufficient.

Standards must have been in force at the time of the summary analysis. Standards scheduled to come into force on a future date were included in the summary table only.

The level of enforceability of the standards or lack thereof was not considered as a criterion for inclusion in the summary analysis.

ISO: International Organization for Standardization.

### Data collection

We extracted the following data items, if found: definitions of PM_2.5_; absolute PM_2.5_ concentration limits; averaging periods to which absolute PM_2.5_ concentration limits apply (e.g. 20 minutes, 24 hours, annual); averaging method (e.g. arithmetic mean, 98th or 99th percentile); envelope averaging period (e.g. 3 years for the 24-hour standard); minimum legally mandated number of valid data points (e.g. 75%); number of permitted exceedances of the PM_2.5_ limit over the averaging period (e.g. nine days per year); tiers of standards (e.g. commercial and residential, primary and secondary); categories of standards (e.g. enforced, voluntary or target); and dates from which standards were effective. We also identified separate standards for some subnational or supranational jurisdictions. 

We obtained the data on population and area of jurisdictions from the World Population Review^7^, and the data on country estimates for mean PM_2.5_ ambient concentrations for 2016 from the World Health Organization (WHO).^8^ These WHO data are synthesized from the data routinely measured at selected stationary monitoring stations in urban areas, satellite remote sensing, topography and population estimates. 

The data on the standards were initially compiled by one author in 2018 and 2019 and were independently verified and updated in September 2019 against the sources by another author to ensure accuracy, except for Egypt, interpreted by a colleague and native speaker. We later updated and reanalysed the standards effective in July 2020.

We converted the Minguo calendar dates in China, Taiwan’s regulations to the Roman calendar. 

### Data analysis

We made a descriptive analysis of how the metrics of the standards compared across different jurisdictions. We analysed the standards against the total population of jurisdictions, population density and geographical area of jurisdictions. We also compared the standards against the levels of actual urban PM_2.5_ air pollution in different jurisdictions to determine where the standards were met and where they were exceeded. 

We categorized the PM_2.5_ air quality standards as: (i) enforced, when a penalty, enforcement, compliance or a similar term was mentioned in the source; (ii) voluntary, when stated so in the source; or (iii) target, when a policy statement existed regarding a level of PM_2.5_ that various stakeholders agreed to work to achieve. We provide this classification to illustrate the approximate relative occurrence of the three different regulatory approaches. This classification should be interpreted with caution because stakeholders in each jurisdiction may by law or in reality apply differing interpretations of regulatory statements regarding enforcement or lack thereof.

## Results

We identified the existence of PM_2.5_ ambient air quality standards in 62 subnational, national and supranational jurisdictions worldwide, including 58 countries. The analysed national and regional PM_2.5_ ambient air quality standards are listed in Table 1 (available at: http://www.who.int/bulletin/volumes/99/2/19-245704). We obtained data on actual PM_2.5_ ambient air pollution for 175 national jurisdictions. Out of these, we used the data on actual PM_2.5_ ambient air pollution for 57 jurisdictions for the analyses of PM_2.5_ ambient air quality standards versus ambient PM_2.5_ air pollution.

**Table 1:** Air quality standards for the concentration of PM_2.5_ around the world, effective before July 2020

Area or jurisdiction by WHO region	PM_2.5_ standard, current	Since year	PM_2.5_ standard, future (year)	Enforced, voluntary or target^a^	Reference(s)
**Global**					
**WHO guidelines**					
Level of no health effects	3–5 µg/m^3^	NA	NA	NA	WHO, 2006^9^
Target levels	Annual: 10 µg/m^3^; 24-hour: 25 µg/m^3^	2005	Plans not published	NA	WHO, 2006^9^
**African Region**					
South Africa	Annual: 20 µg/m^3^; 24-hour: 40 µg/m^3^	2016	Annual: 15 µg/m^3^; 24-hour: 25 µg/m^3^ (2030)	Enforcement regulations in draft stage	Department of Environmental Affairs of the Government of South Africa, 2012^10^
**Region of the Americas**					
Argentina, Buenos Aires	Annual: 15 µg/m^3^; 24-hour: 65 µg/m^3^	NR	Plans not published	NR	The Clean Air Institute, 2012^11^
Bolivia, La Paz	Annual: 10 µg/m^3^; 24-hour: 25 µg/m^3^	NR	Plans not published	NR	The Clean Air Institute, 2012^11^
Canada	Annual: 8.8 µg/m^3^ (3-year average of the annual average of all 1-hour concentrations); 24-hour: 27 µg/m^3^ (3-year average of the annual 98th percentile of the daily 24-hour average concentrations)	2020	Plans not published	Voluntary	Canadian Council of Ministers of the Environment, 2020^12^
Canada, Province of Quebec	24-hour: 30 µg/m^3^	2011	Plans not published	Voluntary	Ministry of the Environment and the Fight against Climate Change, 2016^13^
Canada, Province of Ontario	24-hour: 30 µg/m^3^ (3-year average of the annual 98th percentile of the daily 24-hour average concentrations); 24-hour: 25 µg/m^3^ for individual sources	2012	Plans not published	Voluntary	Standards Development Branch of the Ontario Ministry of the Environment, 2012^14^
Chile	Annual: 20 µg/m^3^ (98th 1-year percentile); 24-hour: 50 µg/m^3^ (3-year average)	2011	Plans not published	Target	Ministry of the Environment of Chile, 2011^15^
Colombia	Annual: 25 µg/m^3^; 24-hour: 50 µg/m^3^	NR	Plans not published	NR	The Clean Air Institute, 2012^11^
Dominican Republic	Annual: 15 µg/m^3^; 24-hour: 65 µg/m^3^	NR	Plans not published	NR	The Clean Air Institute, 2012^11^
Ecuador	Annual: 15 µg/m^3^; 24-hour: 65 µg/m^3^	NR	Plans not published	NR	The Clean Air Institute, 2012^11^
El Salvador	Annual: 15 µg/m^3^; 24-hour: 65 µg/m^3^	NR	Plans not published	NR	The Clean Air Institute, 2012^11^
Mexico	Annual: 12 µg/m^3^ (average of 24-hour concentrations over at least 1 year; at least 75% of 24-hour samples must be valid in each of 4 quarters of the year); 24-hour: 45 µg/m^3^ (arithmetic mean with at least 75% of valid hourly concentrations, 18 records)	2014	Plans not published	Target	Secretary of Health of the United Mexican States, 2014^16^
Paraguay	Annual: 15 µg/m^3^; 24-hour: 30 µg/m^3^	2015	Plans not published	NR	Kutlar Joss et al., 2017^17^
Peru	Annual: 15 µg/m^3^; 24-hour: 25 µg/m^3^	2014	Plans not published	NR	The Clean Air Institute, 2012^11^
Trinidad and Tobago	Annual: 15 µg/m^3^; 24-hour: 65 µg/m^3^	2015	Plans not published	NR	Kutlar Joss et al., 2017^17^
United States of America	Annual, primary (protective of public health): 12 µg/m^3^; Annual, secondary (protective of public welfare): 15 µg/m^3^; 24-hour: 35 µg/m^3^ (98th percentile averaged over 3 years)	2012 (24-hour: value set in 2006, kept in 2012)	Plans not published	Enforced	United States Environmental Protection Agency, 2013;^18^ United States Environmental Protection Agency, 2016^19^
**South-East Asia Region**					
Bangladesh	Annual: 15 µg/m^3^; 24-hour: 65 µg/m^3^	2005	Plans not published	Target (long-term objective)	Asian Development Bank and the Clean Air Initiative for Asian Cities Center, 2006^20^
India	Annual: 40 µg/m^3^; 24-hour: 60 µg/m^3^ (98th 1-year percentile)	2009	Plans not published	Enforced	Central Pollution Control Board of the Ministry of Environment, Forest and Climate Change of the Government of India, 2009^21^
**European Region**					
European Union Member States (28 countries) and Ukraine	Annual: 25 µg/m^3^; 24-hour: none; Average exposure indicator: 20 µg/m^3^	2015	All measures to reach 18 µg/m^3^, average exposure indicator (2020)	Enforced	European Commission, 2017;^22^ Association of Engineers-Consultants of Ukraine, 2015^23^
Norway	Annual: 12 µg/m^3^; 24-hour: none	2015	Plans not published	NR	Norwegian Environment Agency, 2012^24^
Russian Federation	Annual: 25 µg/m^3^; 24-hour: 35 µg/m^3^ (99th annual percentile); 20-minute: 160 µg/m^3^	2010	Plans not published	Enforced	Chief Government Sanitary Physician of the Russian Federation, 2018^25^
Switzerland	Annual: 10 µg/m^3^ (arithmetic mean)	2018	Plans not published	Enforced	The Swiss Federal Council, 2018^26^
**Eastern Mediterranean Region**					
Egypt	Annual: 50 µg/m^3^; 24-hour: 80 µg/m^3^	2012	Plans not published	NR	Egyptian Environmental Affairs Agency of the Ministry of Environment of the Arab Republic of Egypt, 2012^27^
Pakistan	Annual: 15 µg/m^3^; 24-hour: 35 µg/m^3^ (98th 3-year percentile)	NR	Plans not published	NR	Asian Development Bank and the Clean Air Initiative for Asian Cities Center, 2006;^28^ Niaz et al., 2016^29^
Saudi Arabia	Annual: 15 µg/m^3^; 24-hour: 65 µg/m^3^ (exceedances of either standard “as a result of abnormal natural background concentrations shall not be considered a violation of the standard”)	2001	Plans not published	NR	Royal Commission for Jubail and Yanbu, 2004^30^
**Western Pacific Region**					
Australia	Annual: 8 µg/m^3^; 24-hour: 25 µg/m^3^	NR	Plans not published	Enforced	Department of the Environment and Heritage of the Australian Government, 2005^31^
China	First-class zone (residential) Annual: 15 µg/m^3^; 24-hour: 35 µg/m^3^ Second-class zone (commercial) Annual: 35 µg/m^3^; 24-hour: 75 µg/m^3^	2016	Plans not published	Enforced	Ministry of Environmental Protection of the People's Republic of China, 2016^32^
China, Taiwan	Annual: 15 µg/m^3^; 24-hour: 35 µg/m^3^	2012, Minguo calendar 101	Annual: 15 µg/m^3^ (2020, Minguo calendar 109)	Enforced	Environmental Protection Administration Executive Yuan Republic of China, 2015^33^
China, Hong Kong SAR	Annual: 35 µg/m^3^; 24-hour: 75 µg/m^3^ (with 9 exceedances allowed)	2014	Plan to reduce emissions to achieve 2014 standard	Target	Environmental Protection Department of the Government of the Hong Kong SAR, 2017;^34^ Environment Bureau, 2013^35^
Japan	Annual: 15 µg/m^3^; 24-hour: 35 µg/m^3^ (98th annual percentile)	2009	Plans not published	NR	Ministry of the Environment, Government of Japan, 2009^36^
Republic of Korea	Annual: 20 µg/m^3^; 24-hour: 50 µg/m^3^ (98th annual percentile)	2015	Annual: 15 µg/m^3^ (2030)	Enforced	Ministry of Environment of the Republic of Korea, 2017;^37^ Ministry of Environment of the Republic of Korea, 2017;^38^ Ministry of Environment of the Republic of Korea, 2015;^39^ Shin, 2016^40^
Singapore	Annual: 12 µg/m^3^; 24-hour: mean 37.5 µg/m^3^	2020	Annual: 10 µg/m^3^ (long-term); 24-hour: mean 25 µg/m^3^ (long-term)	Target	Ministry of the Environment and Water Resources of the National Environment Agency of Singapore, 2015;^41^ National Environment Agency of the Singapore Government, 2017^42^
Viet Nam	Annual: 25 µg/m^3^; 24-hour: 50 µg/m^3^	NR	Plans not published	NR	Ministry of Natural Resources and Environment of Viet Nam, 2013^43^

NA: not applicable; NR: not reported or no information available; PM: particulate matter; SAR: Special Administrative Region; WHO: World Health Organization.

^a^ We classified standards as enforced when a penalty, enforcement, compliance or a similar term was mentioned in the source; voluntary when stated so in the source; or target when a policy statement existed regarding a level of PM_2.5_ that various stakeholders agreed to work to achieve.

Note: PM_2.5_ is mass concentration of aerosol particles smaller than approximately 2.5 μm.^3^

### Averaging periods for measurements

Different jurisdictions set different intervals over which they average the measured PM_2.5_ concentrations, such as 20 minutes, 24 hours, annual and 3 years. Most jurisdictions used the 98th or 99th percentile, and some used the arithmetic mean of all PM_2.5_ measurements over a prescribed period. For example, in the USA, the annual arithmetic mean is used in the annual PM_2.5_ standard, and the 98th percentile of 24-hour arithmetic means of concentrations over a 3-year period is used in the 24-hour PM_2.5_ standard. In the Russian Federation, the 99th percentile of 24-hour arithmetic means of concentrations over 1 year is applied. Some jurisdictions set a maximum allowed number of exceedances of a time-averaged PM_2.5_ concentration. For example, nine exceedances per year are allowed in Hong Kong Special Administrative Region (SAR), and no exceedances are allowed in the Russian Federation. Critically, many jurisdictions did not specify any averaging method, the minimum percentage of valid data points, or exceedances.

### Stringency of air quality standards

**Fig. 2:**
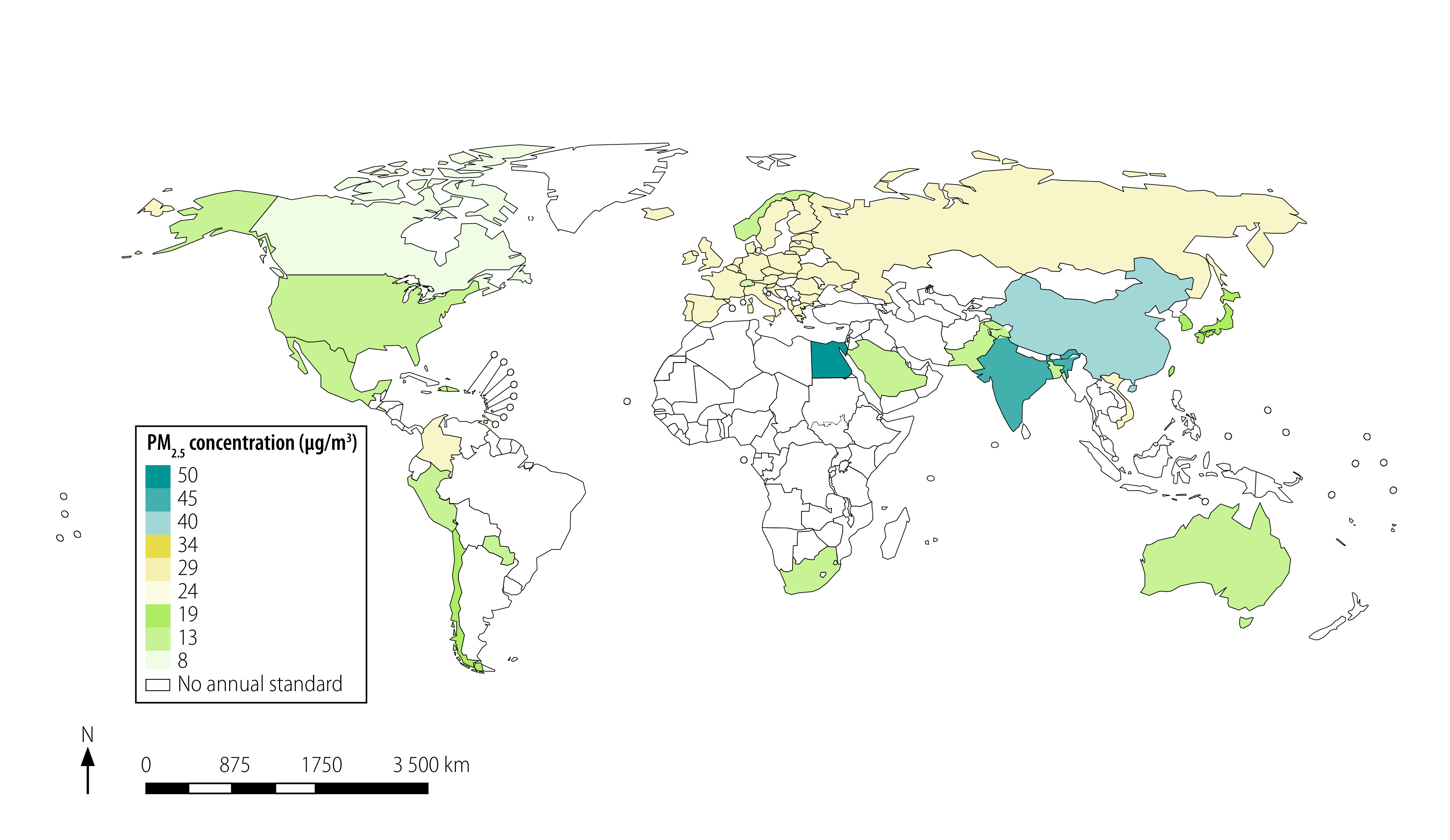
Annual ambient PM_2.5_ air quality standards worldwide

**Fig. 3:**
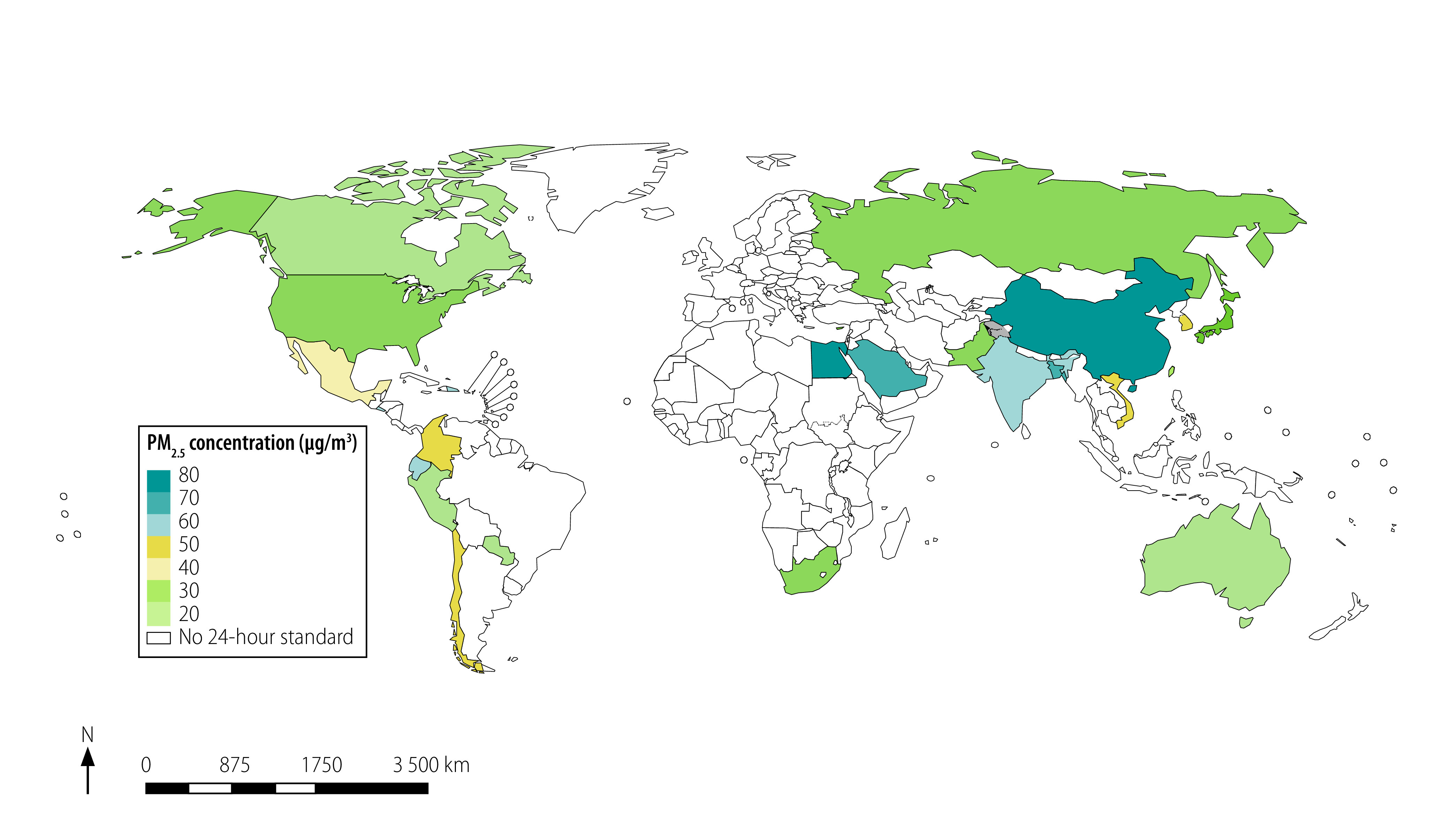
24-hour ambient PM_2.5_ air quality standards worldwide

**Fig. 4:**
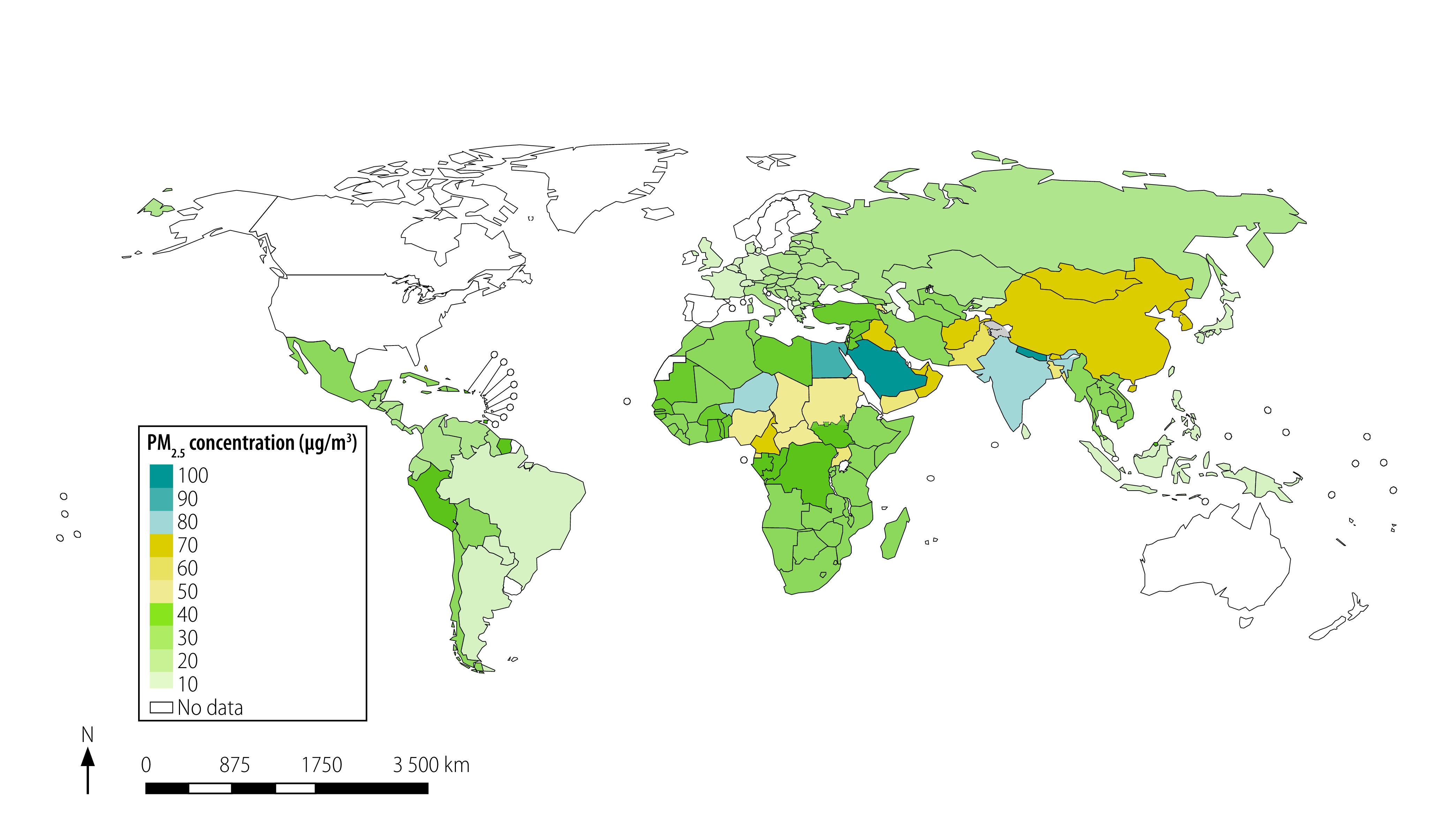
Jurisdictions where annual PM_2.5_ ambient air pollution meet or exceeded WHO guidelines

Fig. 2 and Fig. 3 present a map of the world with jurisdictions coloured according to the stringency of the annual and 24-hour standards. For China, we used the commercial-area PM_2.5_ standards because many people lived near factories and other sources of air pollution. The existing annual standards ranged from 8 to 75 µg/m^3^ in different countries worldwide (Fig. 2). Therefore, most annual standards exceeded both the level at which no detected health effects are expected according to WHO (3–5 µg/m^3^) and the guideline annual PM_2.5_ pollution limits set by WHO. These guidelines are 10 µg/m^3^ (annual) and 25 µg/m^3^ (24-hour).^9^ The real ambient air pollution also exceeded WHO guidelines in most of the world (Fig. 4).

PM_2.5_: mass concentration of aerosol particles smaller than approximately 2.5 μm.

Note: Data for China are the commercial PM_2.5_ standard.

PM_2.5_: mass concentration of aerosol particles smaller than approximately 2.5 μm.

Note: Data for China are the commercial PM_2.5_ standard.

PM_2.5_: mass concentration of aerosol particles smaller than approximately 2.5 μm; WHO: World Health Organization.

Notes: World Health Organization guideline annual PM_2.5_ pollution limit is 10 µg/m^3^. Data on PM_2.5_ ambient air pollution are from World Health Organization, 2016.^8^

Fewer jurisdictions had PM_2.5_ 24-hour standards than annual standards. Notably, only the Russian Federation had a 24-hour standard in the European Region. The Russian Federation had a 20-minute PM_2.5_ standard along with the 24-hour and annual standards, while most other countries of the former Soviet Union did not have any PM_2.5_ standards.

In the USA, there were primary and secondary standards. This primary standard allows for an adequate safety margin to protect public health, considering the uncertainties of available technical and scientific information. The secondary standard has no attainment deadline and is based on known or anticipated adverse effects on public welfare, including ecosystems, buildings and monuments.^18^

In the EU countries, additional PM_2.5_ objectives targeted population exposure to fine particles. These objectives are set at the national level and based on the average exposure indicator, which is a 3-year running annual mean PM_2.5_ concentration averaged over selected monitoring stations in urban areas (Table 1).^44^ Ukraine, which has an association agreement with the EU, adopted the EU’s PM_2.5_ standard to take effect in 2018. The EU supported the creation of the air quality monitoring infrastructure and implementation of the standard in Ukraine since 2015, yet progress has been slow, and the monitoring network has not been completed as of 2020.^45^

In the Eastern Mediterranean Region, with known high levels of PM_2.5_ air pollution due to desert dust, fuel-burning emissions and oil refining, only Egypt, Pakistan and Saudi Arabia had PM_2.5_ air quality standards.^46^^,^^47^

South Africa was the only country in the African Region with a PM_2.5_ standard. The current annual standard of 20 µg/m^3^ and the 24-hour standard of 40 µg/m^3^ will be lowered to 15 µg/m^3^ and 25 µg/m^3^, respectively, on 1 January 2030.^10^

China used different PM_2.5_ standards for the first-class (residential) and the second-class (commercial) zones. Both the annual and the 24-hour standards differed substantially for the two zones: 15 µg/m^3^ annual and 35 µg/m^3^ 24-hour for the first-class zones and 35 µg/m^3^ annual and 75 µg/m^3^ 24-hour for the second-class zones.

### Air quality standards by population density

**Fig. 5:**
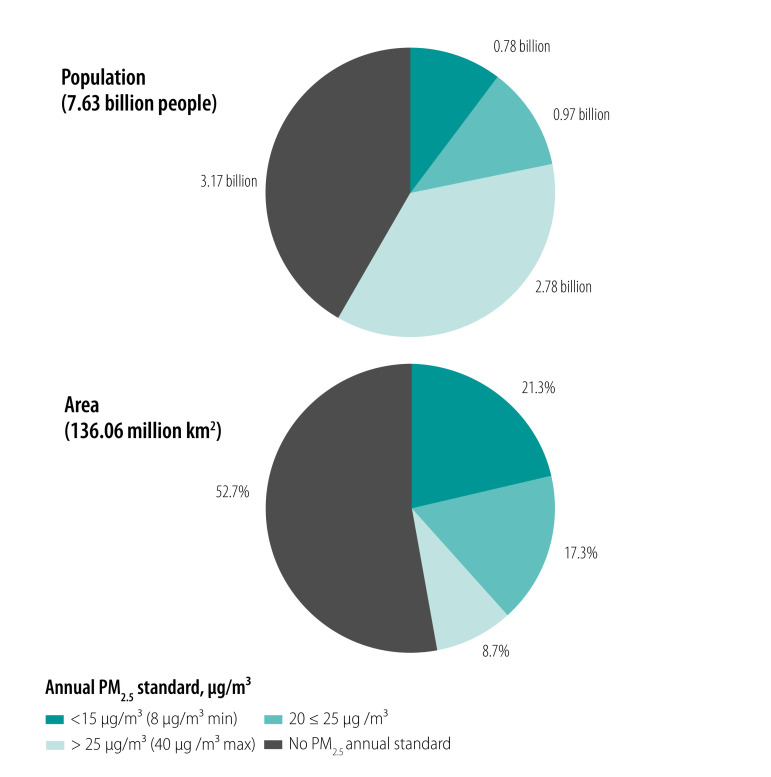
Population and total area covered by different annual PM_2.5_ ambient air quality standards worldwide

Of the world’s total area of jurisdictions in the WHO World Population Review (136.06 million km^2^), just under half (64.36 million km^2^; 47.3%) was part of national jurisdictions with any PM_2.5_ annual ambient air quality standard (Fig. 5). The medium-stringency annual standards ≤ 25 µg/m^3^ covered 52.52 million km^2^ or 38.6% of the world’s total area of national jurisdictions, including 28.98 million km^2^ or 21.3% protected by the strictest official annual PM_2.5_ ambient air quality standards ≤ 15 µg/m^3^. The least stringent annual standards exceeding 25 µg/m^3^ (up to 40 µg/m^3^ in India) covered only 11.84 million km^2^ or 8.7% of the world land part of national jurisdictions, home to 2.78 billion people or 36.6% of the global population of 7.63 billion in 2018.^7^ Areas where no PM_2.5_ ambient air quality standard was in effect are home to 3.17 billion people. 

PM_2.5_: mass concentration of aerosol particles smaller than approximately 2.5 μm.

Source: World Population Review, 2019.^7^

**Fig. 6:**
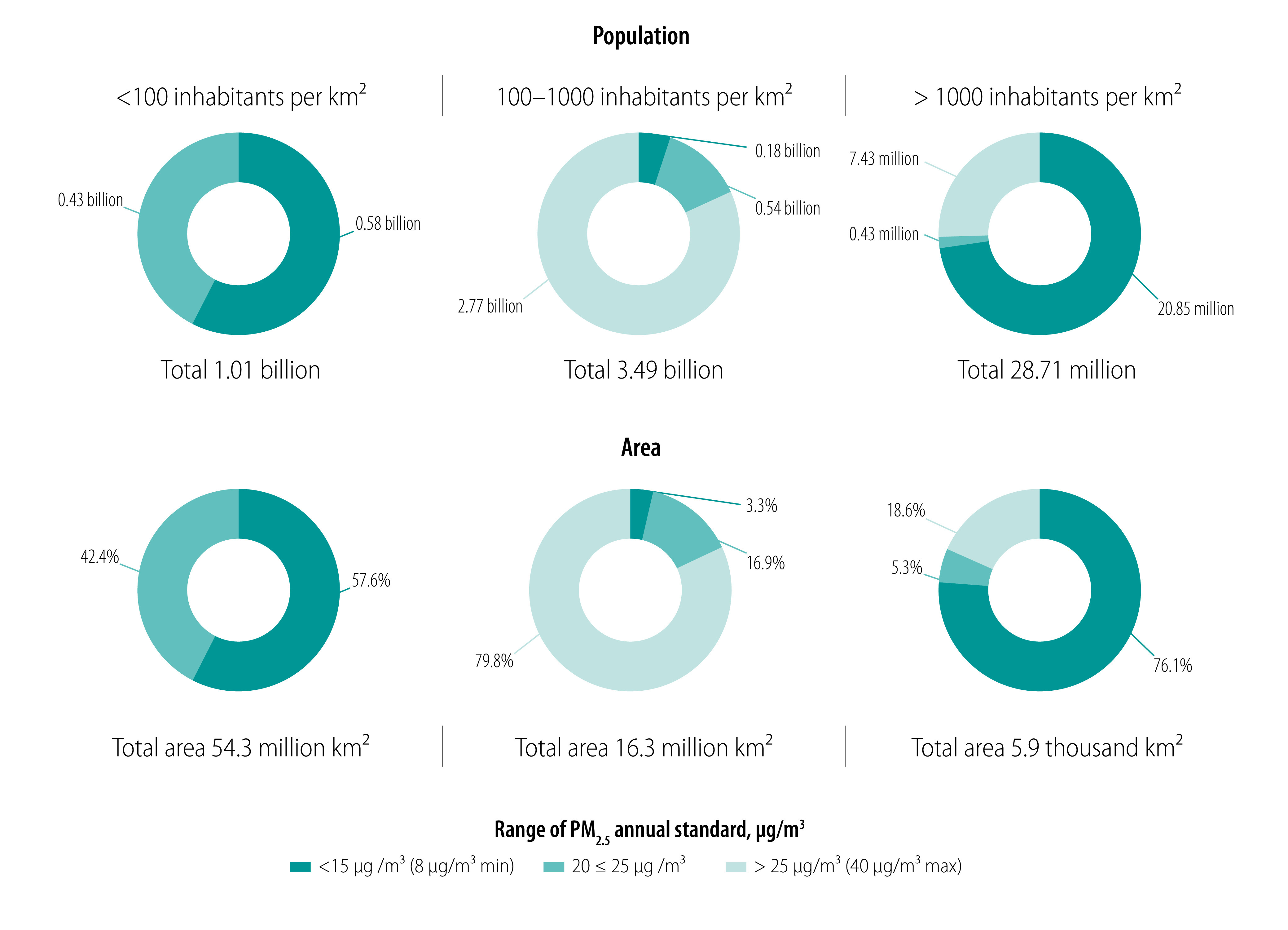
Analysis of total population and total area of jurisdictions where different annual PM_2.5_ ambient air quality standards are in effect worldwide by population density

We compared the total population and area of jurisdictions by annual PM_2.5_ standard and population density (Fig. 6). The areas of low population density (< 100 inhabitants per km^2^) applied only the strictest (≤ 15 µg/m^3^) or medium (20–25 µg/m^3^) annual PM_2.5_ standards. In the areas of high population density of 100–1000 inhabitants per km^2^, most people and land were covered by the least stringent annual PM_2.5_ standards (> 25 µg/m^3^). However, in areas with the highest population density (> 1000 inhabitants per km^2^) with a PM_2.5_ ambient air quality standard, most population and land were covered by the strictest standards (≤ 15 µg/m^3^). Therefore, high population density alone cannot be a barrier to achieving compliance with stringent standards. Many densely populated cities within sparsely populated jurisdictions were covered by and often met the strictest standards set by those jurisdictions.

PM_2.5_: mass concentration of aerosol particles smaller than approximately 2.5 μm.

**Fig. 7:**
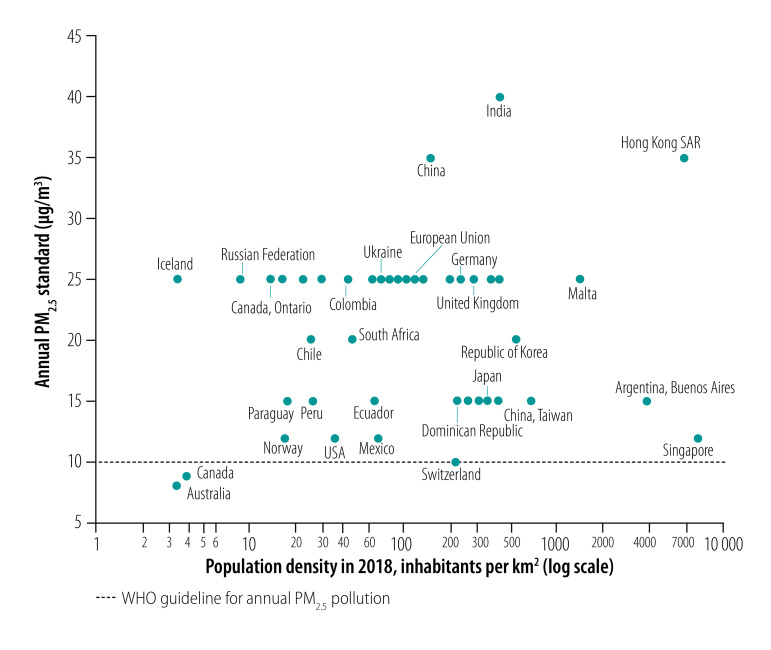
Annual PM_2.5_ ambient air quality standards and population density worldwide

We plotted annual PM_2.5_ standards in individual jurisdictions listed in Table 1 versus the population density (logarithmic scale), including individual EU’s national jurisdictions (Fig. 7). Several notable clusters of jurisdictions stood out. Australia and Canada had a combination of very strict annual PM_2.5_ ambient air quality standards (8 and 8.8 µg/m^3^, respectively) and low population density (3.3 and 3.7 inhabitants per km^2^, respectively), but contained several densely populated cities. Singapore had one of the highest population densities (8265 inhabitants per km^2^) yet one of the lowest annual PM_2.5_ ambient air quality standards (12 µg/m^3^). Hong Kong SAR also had one of the highest population densities (6785 inhabitants per km^2^), but, unlike Singapore, one of the least stringent annual PM_2.5_ standards (35 µg/m^3^). Both China and India had one of the least stringent annual PM_2.5_ standards in the world (35 and 40 µg/m^3^, respectively) combined with high but different population densities (146 and 416 inhabitants per km^2^). Norway and Paraguay stood out with their stricter annual PM_2.5_ standards (15 µg/m^3^ in both) and low population densities (16.7 and 17.2 inhabitants per km^2^) relative to those in their respective regions. The EU’s annual PM_2.5_ ambient air quality standard was relatively lax among the prosperous jurisdictions, notably higher than in Australia, Canada, Japan, Singapore, South Africa and the USA. Several densely populated jurisdictions could maintain relatively strict annual PM_2.5_ ambient air quality standards: Dominican Republic, El Salvador, Japan, Singapore, China (Taiwan only) and Trinidad and Tobago.

PM_2.5_: mass concentration of aerosol particles smaller than approximately 2.5 μm; SAR: Special Administrative Region; WHO: World Health Organization.

Note: Selected jurisdictions are labelled. The countries adhering to the European Union standard had the annual standard 25 µg/m^3^. World Health Organization guideline annual PM_2.5_ pollution limit is 10 µg/m^3^.

### Comparison of air quality to standards

**Fig. 8:**
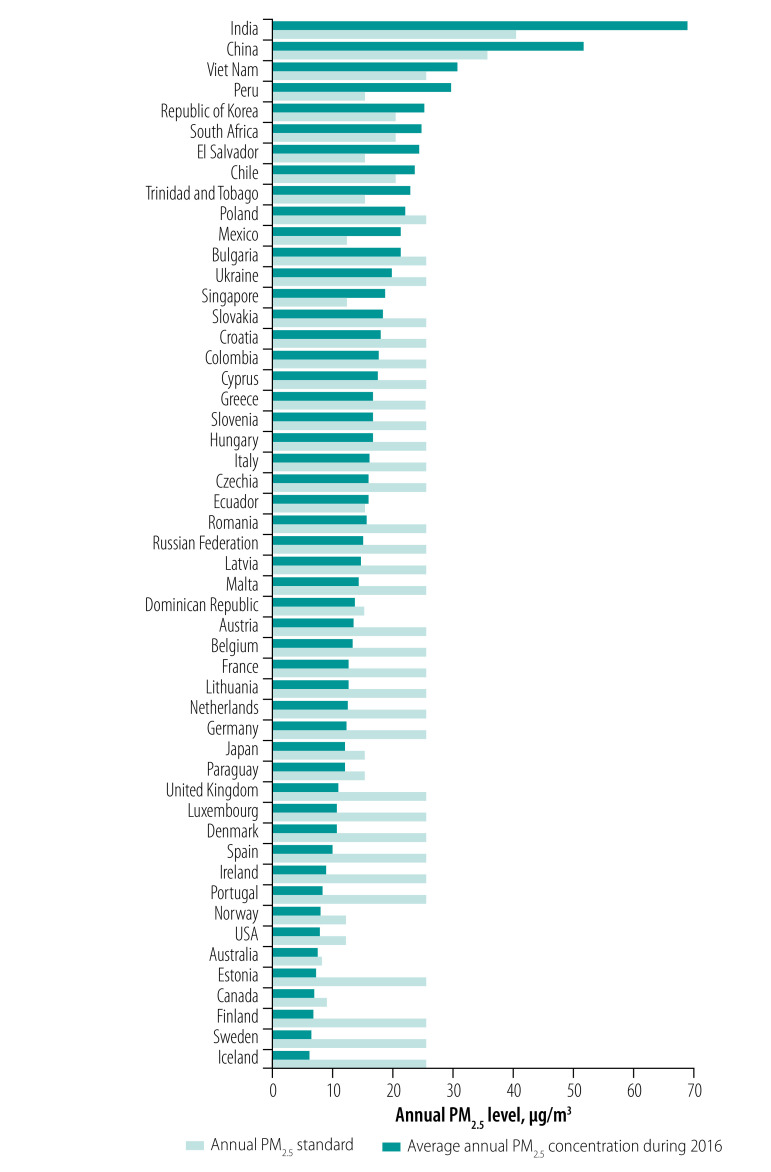
Annual mean PM_2.5_ ambient concentrations worldwide

The annual PM_2.5_ ambient air quality standards were often exceeded in the jurisdictions with the highest PM_2.5_ ambient air pollution (Fig. 8; available at: http://www.who.int/bulletin/volumes/99/2/19-245704). Singapore stood out by its relatively strict annual PM_2.5_ standard despite PM_2.5_ air pollution that considerably exceeded the standard. Where the EU’s standard was in effect, the PM_2.5_ air pollution was highly variable, ranging from 20.8 µg/m^3^ in Bulgaria to 5.9 µg/m^3^ in Iceland.

PM_2.5_: mass concentration of aerosol particles smaller than approximately 2.5 μm.

Notes: The data are for the jurisdictions for which both the mean PM_2.5_ concentrations and the annual PM_2.5_ ambient air quality standards were available. The data are population-weighted for urban populations in the jurisdictions.

**Fig. 9:**
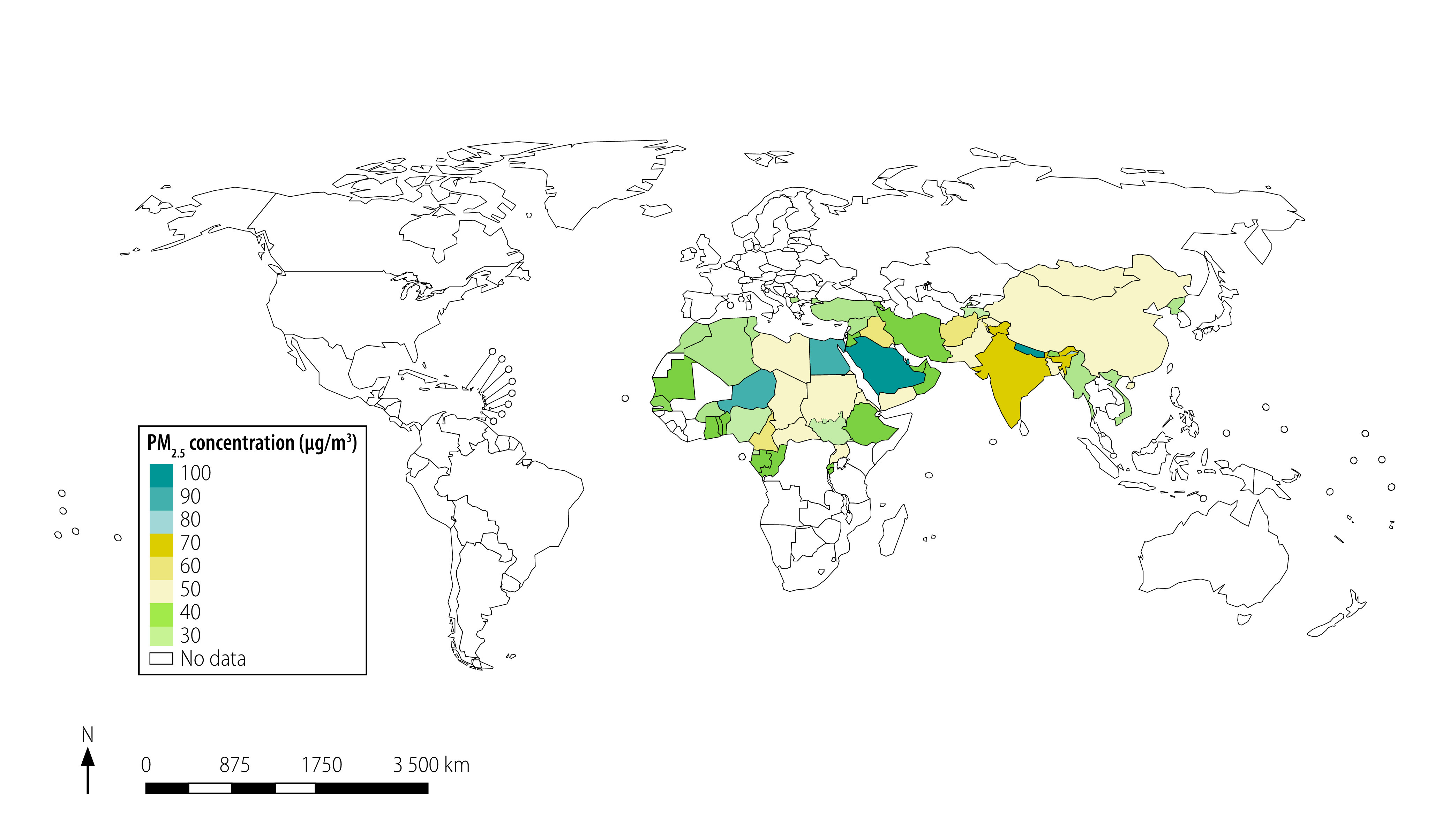
Jurisdictions where annual PM_2.5_ ambient air pollution exceeded 30 µg/m^3^, 2016

We excluded many jurisdictions where PM_2.5_ pollution exceeded 30 µg/m^3^ (Fig. 9) from the analysis because they lacked an annual PM_2.5_ ambient air quality standard. These jurisdictions need urgent PM_2.5_ air pollution reduction measures. These excluded jurisdictions included Armenia, Mongolia, Nepal, North Macedonia, Tajikistan and Turkey and many countries in the African and Eastern Mediterranean Regions.

PM_2.5_: mass concentration of aerosol particles smaller than approximately 2.5 μm.

Note: Data on PM_2.5_ ambient air pollution are from World Health Organization, 2016.^8^

## Discussion

In many jurisdictions, air quality regulations defined PM_2.5_ as all particles smaller than 2.5 μm. This definition does not match the definition published by the International Organization for Standardization (ISO).^3^ Many regulatory documents referred simply to particle diameter rather than aerodynamic diameter, even though the definition of particle diameter as aerodynamic diameter is critical to the ISO definition of PM_2.5_. Various metrics exist for particle diameter besides aerodynamic diameter (detailed list in data repository).^6^^,^^48^^,^^49^ Therefore, regulations referring only to particle diameter without defining it introduce ambiguity. Jurisdictions can solve the problem by updating regulations with references to aerodynamic diameter specifically.

Some jurisdictions used a two-tier system of standards, such as different standards for commercial versus residential areas. One example of such a two-tier system is China, where a laxer standard was used in commercial zones where air pollution levels are generally higher, even though many people live next to China’s factories. Geographically uniform standards are more useful for protecting occupational and public health. However, China's current zone-based system may better protect vulnerable populations, such as children and the elderly in the residential zones, in a time of transition towards a geographically uniform standard.

Jurisdictions within nations may set subnational standards that are weaker than national standards. Canada is one example. The federal PM_2.5_ air quality standard was 8.8 µg/m^3^ (annual) and 27 µg/m^3^ (24-hour). Quebec and Ontario had their own 24-hour PM_2.5_ standards of 30 µg/m^3^, which prevailed over the federal standard. Quebec did not sign on to the federal annual PM_2.5_ standard. However, because air quality standards in Canada are voluntary and overwhelmingly met, no conflict exists.

Short-term standards, such as the 20-minute 160 µg/m^3^ PM_2.5_ standard in the Russian Federation, could be used in parallel with the annual and the 24-hour standards to reveal acute short-term spikes of PM_2.5_ concentrations. The use of such a short-term averaging period, but only when combined with an adequately strict PM_2.5_ concentration limit, can be useful in light of the current knowledge from controlled-exposure research on healthy adults that short-term exposures to high PM_2.5_ concentrations can cause adverse health effects.^50^^,^^51^

The PM_2.5_ fraction contributes the most to the total burden of disease from particulate air pollution exposure.^4^ In the past, jurisdictions with high ambient PM_2.5_ air pollution saw health and environmental benefits from the implementation of PM_2.5_ ambient air quality standards and measures to reduce PM_2.5_ exposure.^52^^,^^53^ However, many jurisdictions still do not regulate PM_2.5_ air pollution or still have standards that are far from the safer levels based on the evidence from epidemiological studies.^9^ Mechanistic studies found that the chemical composition of inhaled particles influences the biological effects these particles cause upon inhalation.^54^ However, health studies conducted to date have predominantly assessed the impact of the total mass of inhaled PM_2.5_ particles over time, irrespective of PM_2.5_ aerosol composition.^55^ Nevertheless, investing efforts into the total PM_2.5_ air pollution reduction may be more beneficial than regulating different PM_2.5_ air pollution components separately. An exception to this approach might be made in areas with strong natural dust sources, such as the Middle East, where monitoring and controlling anthropogenic source emissions could be more effective.

Standards and air quality monitoring data cannot be accurately compared between different jurisdictions when data collection and processing methods differ (different PM_2.5_ definitions, averaging periods, exceedances, percentiles). The differences in these metrics result in potential discrepancies between PM_2.5_ ambient air pollution levels and the values recorded and used to determine compliance with the standards. Currently, there is no universal set of metrics used in PM_2.5_ ambient air quality standards that would ensure comparability of monitoring data globally. Without a universal metric, the same absolute PM_2.5_ mass concentration limit can permit different levels of PM_2.5_ pollution. The temporal and spatial distributions of the absolute recorded levels of PM_2.5_ ambient air pollution are used in epidemiological studies and health risk assessment, where the differences in metrics can introduce errors. We suggest worldwide harmonization of the metrics of the PM_2.5_ air quality standards to achieve the same averaging methods and exceedance allowances, or phasing out of exceedance allowances. This harmonization of the metrics of the PM_2.5_ air quality standards may be achieved if the WHO guidelines specify a universal PM_2.5_ definition based on aerodynamic diameter, and establish a common averaging and data recording method.

Enforced, target or voluntary standards were used in different jurisdictions. The goal to achieve the target standards is generally political, where accountability between responsible government branches exists. There is no universal enforcement mechanism and no definition of enforcement in the case of target standards. Enforced standards function through the possibility that at least one responsible party will bear potential financial, administrative or other costs resulting from non-compliance. Unless standards are explicitly defined as voluntary, various types of costs of non-compliance are possible. Canada is a notable exception where PM_2.5_ ambient air quality standards were defined as voluntary. The voluntary PM_2.5_ air quality standards in Canada are uniquely associated with a robust, extensive network of air quality monitoring stations registering only rare local exceedances. Outside of this context, voluntary air quality standards may not be justified.

The success of strict ambient air quality standards in several densely populated jurisdictions demonstrates that high population density should not discourage the implementation of PM_2.5_ ambient air pollution reduction measures, including stricter PM_2.5_ ambient air quality standards.

The current 24-hour standards mask sharp PM_2.5_ concentration spikes over short periods of minutes to hours. Jurisdictions with a high temporal variability of PM_2.5_ concentration, such as in India and China, should consider short-term averaging (such as over 20 minutes or 1 hour) along with high percentiles (such as the 98th or 99th) of 1-hour arithmetic means to monitor and reduce short-term PM_2.5_ spikes.

Our study has some limitations. We could not confirm the existence of PM_2.5_ regulations in certain countries with high PM_2.5_ pollution and associated mortality, including Indonesia, Iraq, Myanmar, Nigeria, Sudan, Thailand and Turkey, even though PM_10_ or other standards may be in place and some jurisdictions without an identified standard might be using WHO guidelines. The Islamic Republic of Iran is an example of such a situation. The Iranian government’s environment department stated on their website that they are guided by the PM_2.5_ standards of the United States Environmental Protection Agency (the department could not be reached for comment). We also found recommendations in the government documents of some of these countries regarding the reduction of particulate emissions. Iranian authorities, for example, have recommendations for numerous interventions to reduce emissions, including limits on vehicle emissions, industry, open burning, cooking fuels and enforcement mechanisms. Also some jurisdictions might have had regulations that included PM_2.5_ that were not included in the analysis because they were not defined as PM_2.5_ or were not accessible to the authors due to the language barrier or other difficulties with access to information. Inaccessibility, along with our specific inclusion and exclusion criteria, and our data reflecting the standards in 2020, could have caused slight differences between our results and the WHO maps on air quality standards.^56^

In conclusion, to protect people's health from harmful PM_2.5_ air pollution, we suggest that regulatory agencies and governments adopt and regularly tighten PM_2.5_ ambient air quality standards. Where PM_2.5_ air quality often exceeds WHO guidelines, these standards should be enforced with clearly defined enforcement mechanisms. The standards must be stringent enough for each local level of PM_2.5_ ambient air pollution to drive meaningful air pollution reduction actions that are adequate and meaningful considering the level of PM_2.5_ ambient air pollution in a given jurisdiction. Governments and agencies must avoid using the arithmetic mean metric, which tends to conceal high-pollution episodes reducing governments’ ability to identify and remediate sources of PM_2.5_. We suggest that high percentiles should be used instead of the arithmetic mean.
